# Sex as a Biological Variable in Tissue Engineering and Regenerative Medicine

**DOI:** 10.1146/annurev-bioeng-092222-030857

**Published:** 2023-04-27

**Authors:** Josephine B. Allen, Christopher Ludtka, Bryan D. James

**Affiliations:** 1 Department of Materials Science and Engineering, University of Florida, Gainesville, Florida, USA; 2 J. Crayton Pruitt Family Department of Biomedical Engineering, University of Florida, Gainesville, Florida, USA; 3 Department of Marine Chemistry and Geochemistry, Woods Hole Oceanographic Institution, Woods Hole, Massachusetts, USA; 4 Department of Biology, Woods Hole Oceanographic Institution, Woods Hole, Massachusetts, USA

**Keywords:** precision medicine, sexual dimorphism, sex differences, tissue models, stem cells, sex characteristics

## Abstract

Although sex differences have been noted in cellular function and behavior, therapy efficacy, and disease incidence and outcomes, the adoption of sex as a biological variable in tissue engineering and regenerative medicine remains limited. Furthering the development of personalized, precision medicine requires considering biological sex at the bench and in the clinic. This review provides the basis for considering biological sex when designing tissue-engineered constructs and regenerative therapies by contextualizing sex as a biological variable within the tissue engineering triad of cells, matrices, and signals. To achieve equity in biological sex within medicine requires a cultural shift in science and engineering research, with active engagement by researchers, clinicians, companies, policymakers, and funding agencies.

## 1. INTRODUCTION

**Figure 1:**
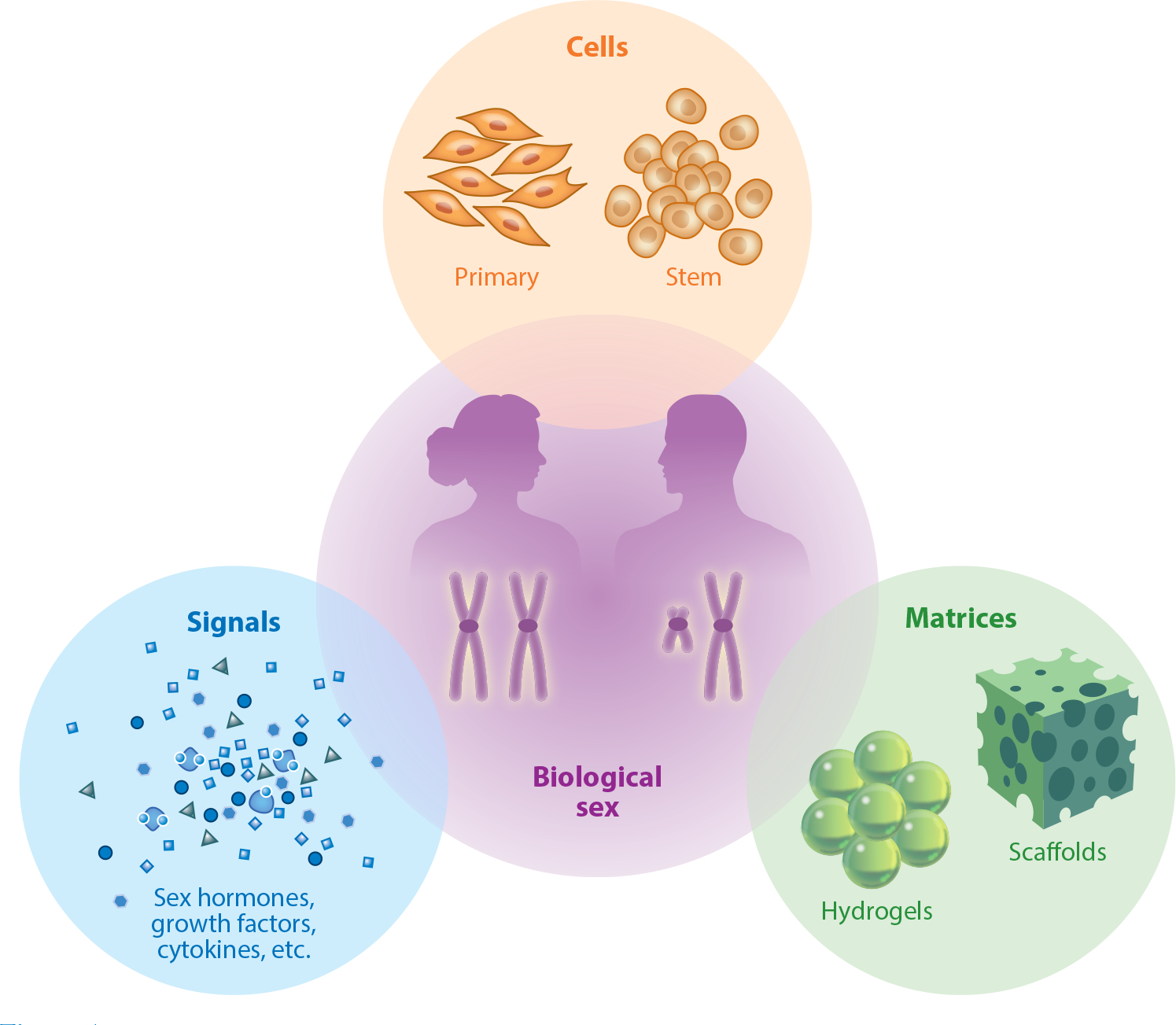
The tissue engineering triad includes a combination of cells cultured on biomaterial matrices with appropriate biophysical and chemical signals, which combine to promote tissue formation and regeneration. Each component of the triad is affected by factors related to biological sex, including sex-based hormones and sex chromosomes.

Regenerative medicine is an interdisciplinary field defined as the process of replacing or regenerating human cells, tissues, or organs to restore or establish healthy function. This approach often includes using a therapeutic cell population, a complementary biomaterial scaffold, and the coordination of appropriate signaling based on tissue engineering principles (Figure 1). Promising preclinical and clinical studies support using regenerative medicine strategies to treat various diseases and dysfunctions, including dermal wounds, cardiovascular diseases and traumas, and certain types of cancers, among many others. While sex-based differences have been documented in the physiology literature for decades, their consideration in the engineering design of regenerative therapies has consistently fallen short. Though the field continues to advance steadily, in recent years, calls to action from the academic community and changes to guidelines from major funding agencies have catalyzed the push to evaluate sex differences and health disparities in biomedical engineering. It behooves researchers to include and consider sex as a biological variable (SABV) from several perspectives: the equitable testing and development of therapies, the treatment of sex differences as a novel subject of interest where historically there has been a paucity of investigation, and the robust and successful design and translation of research from the bench to the clinic.

Concepts of sex and gender are often used interchangeably inside and outside the scientific literature and require clarification, historical context, and a brief discussion. Sex and gender are distinct and not synonymous. According to the US National Academy of Medicine, sex is the classification of living things, generally male or female, according to their reproductive organs and functions assigned by chromosomal complement (1, 2). In contrast, gender refers to a complex psychosocial construct that considers biology, the influences of society, and the environment (2). Sex, gender, and their interaction can influence molecular and cellular processes, clinical characteristics, and health outcomes (3). While we focus on biological sex in this review, it is noteworthy that gender is also an important trait gaining attention. In fact, the Canadian Institutes of Health Research and the European Commission endorsed integrating sex and gender into health research (4, 5). Evidence shows that sex, gender, and intersectional analysis influence health outcomes, and an argument has been made for including broader biological variables in the biomedical sciences and the field of regenerative medicine. In a recent report, Nielsen and colleagues (6) argued for gender as a sociocultural variable to complement SABV in clinical research as a strategy to reveal the influence of gender-related factors on health and disease processes. While this topic is not included in this review, several informative reviews and commentaries discuss the intersection of sex and gender in science and engineering (7–9).

The impacts of biological sex manifest as health disparities, which are well known, well characterized, and the focus of extensive study in the clinical setting. Health disparities in diseases such as heart and vascular disease (10, 11), autoimmune diseases, several cancers (12, 13), some neurological disorders (14), lung disease, liver disease, and others highlight the broad reach of this issue. For example, the prevalence of atherosclerotic cardiovascular disease (CVD) in males is greater than in females until menopause, after which the prevalence of CVD increases rapidly for females and exceeds that of males (15). Similarly, ischemic heart disease affects females disproportionately due to female-specific pathophysiology that differs from male-centric models of the disease used for diagnosis (10). As for cancers, several forms of the disease are more prevalent in either females or males (13). Noteworthy are the high incidences of thyroid cancer in females relative to males and urinary bladder cancer in males relative to females (13). Additionally, autoimmune diseases such as lupus and rheumatoid arthritis disproportionately affect females more than males. There are also disparities in pharmacokinetics with disparate responses to drug treatments. Females experience adverse drug reactions nearly twice as often as males, yet how biological sex contributes to these outcomes is poorly understood (16). This disparity in drug response has resulted in the withdrawal of several drugs from the market due to adverse effects in female patients. Collectively, there are sex-based health disparities in the onset, progression, severity, and treatment outcomes for many diseases and conditions.

The term precision in precision medicine is indicative of individualized or personalized medicine. This area of medicine moves away from a one-size-fits-all approach to treatment. Instead, it focuses on targeted and individualized therapies for the patient by considering their genetics, environment, and lifestyle. In doing so, personalized treatment is anticipated to result in better patient outcomes. When the therapeutic is a biomaterial, our group and others advocate for developing precision biomaterials (17). Biomaterials have great utility in regenerative medicine, and future advanced biomaterials will require innovative designs that can be tailored to the individual patient’s needs. This effort necessitates including SABV in the early stages of biomaterial development and characterization and during early cell-based in vitro studies (18).

Even though sex-based differences have been documented for quite some time, their standing has been elevated only in more recent years following the advent of precision medicine. As early as November 1999,the Institute of Medicine (now the US National Academy of Medicine) formed a Committee on Understanding the Biology of Sex and Gender Differences and produced a report of their findings entitled *Exploring the Biological Contributions to Human Health: Does Sex Matter?* In their report, the committee pieced together the current, at the time, understanding of sex and gender in human health and made recommendations for the biomedical research community to consider SABV (19). In 2016, the US National Institutes of Health (NIH) introduced an SABV mandate as part of a broader initiative to improve the rigor and reproducibility of research funded by the NIH (20). The policy states that grant applications must include both male and female subjects, which includes cells, in experimental design and analysis, with the primary objectives of broadening the general knowledge base and delivering a more refined understanding of how and in whom basic science findings will best translate into clinical applications. During that same period, in 2015, former US president Barack Obama enacted his precision medicine initiative, which is championed by the ongoing NIH *All of Us* research program. The program aims to individualize healthcare and improve our understanding of sex-based differences in medicine, which has historically been underrepresented. These policies are thought to also reduce costs by identifying sex differences earlier in the translational pipeline and improve women’s health outcomes by identifying sex-based differences that may have been missed when assessing the safety and efficacy of therapies (21).

**Figure 2:**
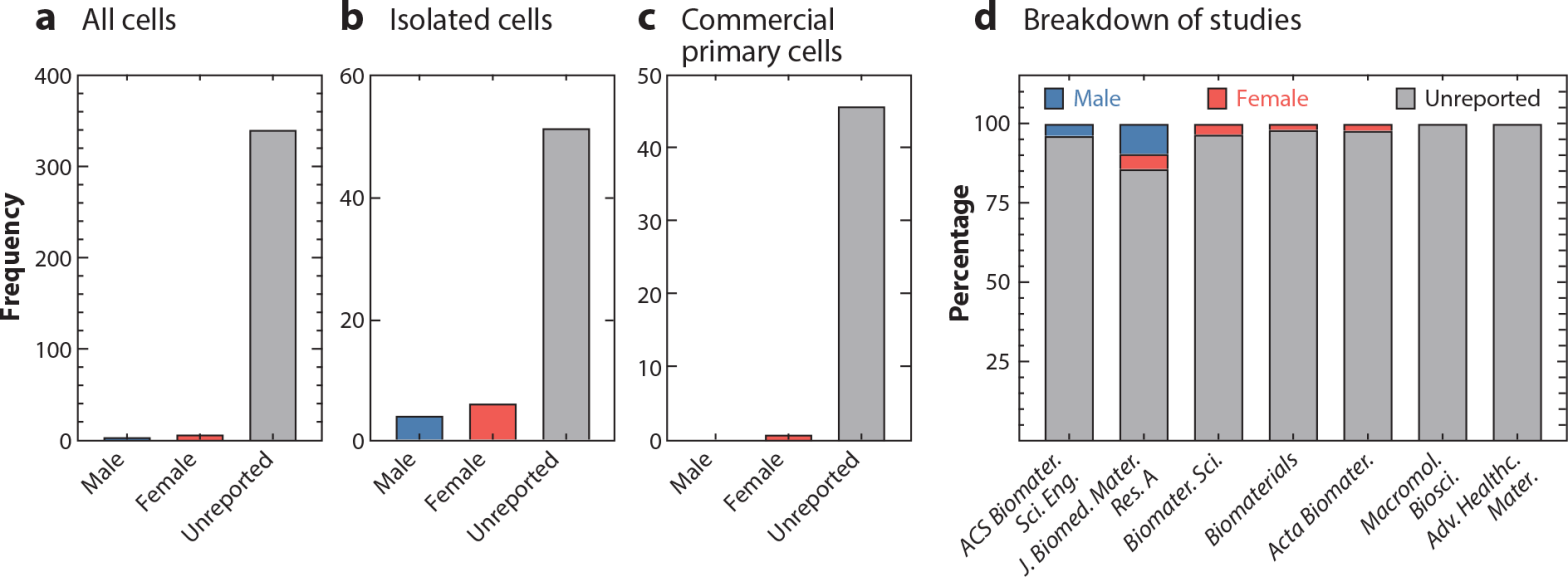
Breakdown of cell sex (*a*) for all the cell culture studies reported, (*b*) for all primary cells isolated or harvested in-house, and (*c*) for all primary cells purchased from commercial vendors. This survey recorded whether the cells used were reported as male or female or if sex was unreported. (*d*) Breakdown of cell sex of all cell culture studies recorded for articles published in each journal in December 2019. Figure adapted with permission from Reference 23; copyright 2021 John Wiley and Sons.

Several years have passed since the NIH mandate and the *All of Us* research program were introduced. As reported in a five-year follow-up, this call to action and the momentum it caused have seen some success and resulted in the discovery of new sex differences across several research fields (22). Although the follow-up evaluation was promising, a recent survey of US researchers conducting animal work in the biomedical field found that SABV has still not been included widely by biomedical researchers (21) nor has in vitro cell sex been reported widely in biomedical and biomaterial peer-reviewed publications (23–26) (Figure 2). Additionally, SABV has not been overwhelmingly adopted within the biomedical engineering community, including in tissue engineering and regenerative medicine research (21). This absence of research presents an opportunity for more advanced strategies and innovative approaches that push biomedical research forward and progress research for broad and diverse populations.

This review serves as a roadmap for biomedical researchers to include SABV in research and engineering design. Rather than summarize an entire field, we focus on the impact of biological sex toward the goals of tissue engineering and regenerative medicine. We acknowledge that this topic is complex and has many variables to consider. This review is organized into three main themes surrounding the tissue engineering triad of cells, matrices, and signals, as each encompasses sex-specific design considerations and biological perspectives (Figure 1). We do not include extensive clinical data or research; instead, we focus on engineering approaches to regenerative medicine. We would be remiss not to acknowledge that, while not included in this review, the intersection between sex, gender, age, ethnicity, genetic ancestry, socioeconomic status, geographic location, disabilities, health, etc., impacts human health outcomes and may contribute to the outcomes of biomedical engineering. To our knowledge, this review is the first to cover this topic in such a manner and serves as one step of many toward more personalized regenerative medicine.

## 2. SEX-SPECIFIC DESIGN CONSIDERATIONS

Tissue engineered systems and regenerative therapies used for precision medicine require a degree of tunability in their properties to meet patient-specific needs. The tunable aspects of a system or therapy should accommodate the inherent differences that form the basis of biological sex. A precise solution requires designing for the many ways in which biological sex pervades the (patho)physiology of cells, matrices, and signals.

### 2.1. Cells

Tissue engineering and regenerative medicine strategies strive for patient cells to be active rather than passive participants in regenerating damaged tissue. This strategy has built on and developed a level of design and practicality around using cells of a specific type (e.g., mesenchymal stem cells, fibroblasts, preosteoblasts, or macrophages), specific species (e.g., mouse, bovine, or human), specific tissue (e.g., pulmonary, umbilical vein, or microvascular endothelial cells), and specific phenotype (e.g., M0, M1, or M2 macrophages) for engineering tissues and therapies. Despite this significant specificity, biological sex has rarely been included in the selection of cells when designing engineered tissues or regenerative therapies. In effect, a level of specificity addressing biological sex has been overlooked. Studies developing organ-on-a-chip technologies, which can be valuable models for diagnostics, drug discovery, and toxicology, have routinely neglected biological sex. Surveys from 2017 and 2022 found that anywhere from 53% to 65% of these devices did not consider biological sex, and of those that did, none analyzed their results on the basis of biological sex (24, 25). The biomaterials community has fared worse. A snapshot of the literature across seven field-specific journals revealed that only ~4% of studies reported cell sex, and none included it as a variable of interest (23). To realize the promise of tissue engineering and regenerative medicine more broadly in the developing age of precision medicine requires acknowledging and accounting for the biological sex of cells.

Fundamental to the distinction between males and females are their pairs of sex chromosomes. Biological males present with XY, and biological females present with XX chromosomes. Though sex chromosomal aneuploidies exist in the general population, albeit infrequently (27), in this review, we consider only female (F) (XX) and male (M) (XY). Redundancy of the X chromosome results in many female-specific chromosomal features, including inactivation and mosaicism. Only one X chromosome is needed for biological function. As a result, one X chromosome undergoes inactivation (X_i_), the process in which one of the two X chromosomes is randomly silenced in each female cell (28). However, some genes (~15%) can escape inactivation, which differs across cell types and individuals (29). Using an engineered aortic valve tissue model, Aguado et al. (30) showed that genes that escape inactivation modulate sex-specific myofibroblast activation with implications for aortic valve stenosis. This work highlights how an in vitro tissue engineered platform can be used to elucidate genetic sex differences (31). Redundancy of the X chromosome is thought to contribute to the female-specific defense against X-linked genetic diseases. X inactivation leads to cellular mosaicism of the active X chromosome (X_a_); thus, only a random fraction of female cells may use an X chromosome that contains a genetic disease, resulting in an apparent resistance to the disease (32). In contrast, male cells are always susceptible to an X-linked genetic disease, if present, because they have only one X chromosome (33). Several immune-related genes (e.g., *IL2RG*, *IL13RA1*, *TLR7*, and *GATA1*) are on the X chromosome, potentiating greater genetic diversity for female immunity (34). Mosaicism is not exclusive to female cells. Male cells may lose their Y chromosome (mLOY) due to environmental conditions (e.g., smoking) and age (e.g., in leukocytes), wherein mLOY can lead to an increased risk of pathogenicity and mortality (35–38). Ultimately, X_i_ and mLOY result in mosaicism of female and male cells, respectively, contributing to innate cell heterogeneity and the potential selection of one phenotype over another depending on environmental conditions. For regenerative medicine, this suggests that during in vitro cell expansion, culture conditions could select one activated X chromosome over another in female cells and contribute to mLOY in male cells (39, 40). Thus, preserving genetic phenotype and karyotype may be equally relevant to engineered tissues as preserving transcriptional and functional phenotype.

Despite having the same types of organs (excepting sex organs), the actual cells and proportions resident to each type of tissue can differ between males and females (41, 42). Notably, this has been described for cardiac tissue; females have a greater proportion of cardiomyocytes (35% F to 25% M) while males have a greater proportion of endothelial cells (25% M to 15% F) (41, 43). Moreover, sex differences in gene expression are largely tissue specific, as revealed by multiorgan, single-cell transcriptomic studies of human tissues (44–46). Some of these differences in gene expression can be intrinsic, being set at birth, while others can be acquired over the natural life span (47). In the context of endothelial cells, intrinsic sex differences in gene expression can include not only sex chromosome linked genes but also autosomal genes (47) as the result of sex-specific genetic variation, epigenetics, and transcriptional machinery (34, 47, 48). In contrast, acquired sex differences in endothelial cell gene expression are largely attributed to regulation by sex hormones (47). Together, these facts imply that tissue engineering and regenerative medicine strategies need to tailor the relative proportion of differentiated cell types according to patient sex. Moreover, the target gene expression profiles of the cells that form an engineered tissue and the mechanistic targets to control gene expression can differ according to biological sex.

Cell-based tissue engineering strategies often use stem cells in pluripotent form or following stimulated differentiation. Principally, stem cells are used and differentiated into target cell types to construct an engineered tissue or provide a therapy. Mature, tissue-specific cells can also be used, whereby environmental cues (matrices and signals) direct them to the desired phenotype. For stem cells, differentiation efficacy and protocols can be sex specific (49–52). For example, reprogramming human amniotic stem cells into induced pluripotent stem cells (iPSCs) by Sendai virus–delivered Yamanaka factors was at least sevenfold more effective for female cells than for male cells by day 21 of reprogramming (51). iPSC differentiation into cardiomyocytes can also be sex specific, with a greater commitment by female iPSCs than male iPSCs toward cardiomyocytes, with underlying mechanisms rooted in X_a_ (49). Comparatively, donor age and ancestry did not influence iPSC commitment for this cell type (49). Additionally, for iPSCs, sex-specific epigenetics, with origins in X_i_ variability, may discount specific female lineages due to their poorer differentiation and upregulation of oncogenes (53). In another instance, the differentiation of human pluripotent stem cells into endothelial progenitor cells by GSK3 inhibition was limited for female cells without adding vascular endothelial growth factor (VEGF) (50). This difference was suggested to be due to the reduced endogenous production of VEGF by female endothelial cells compared with that of males. Accordingly, tissue engineered systems need to consider and ensure that their mechanism for cell differentiation is robust and tailorable to the differentiation needs of both male and female cells to produce a functional tissue that will be successful in the individualized patient.

**Figure 3:**
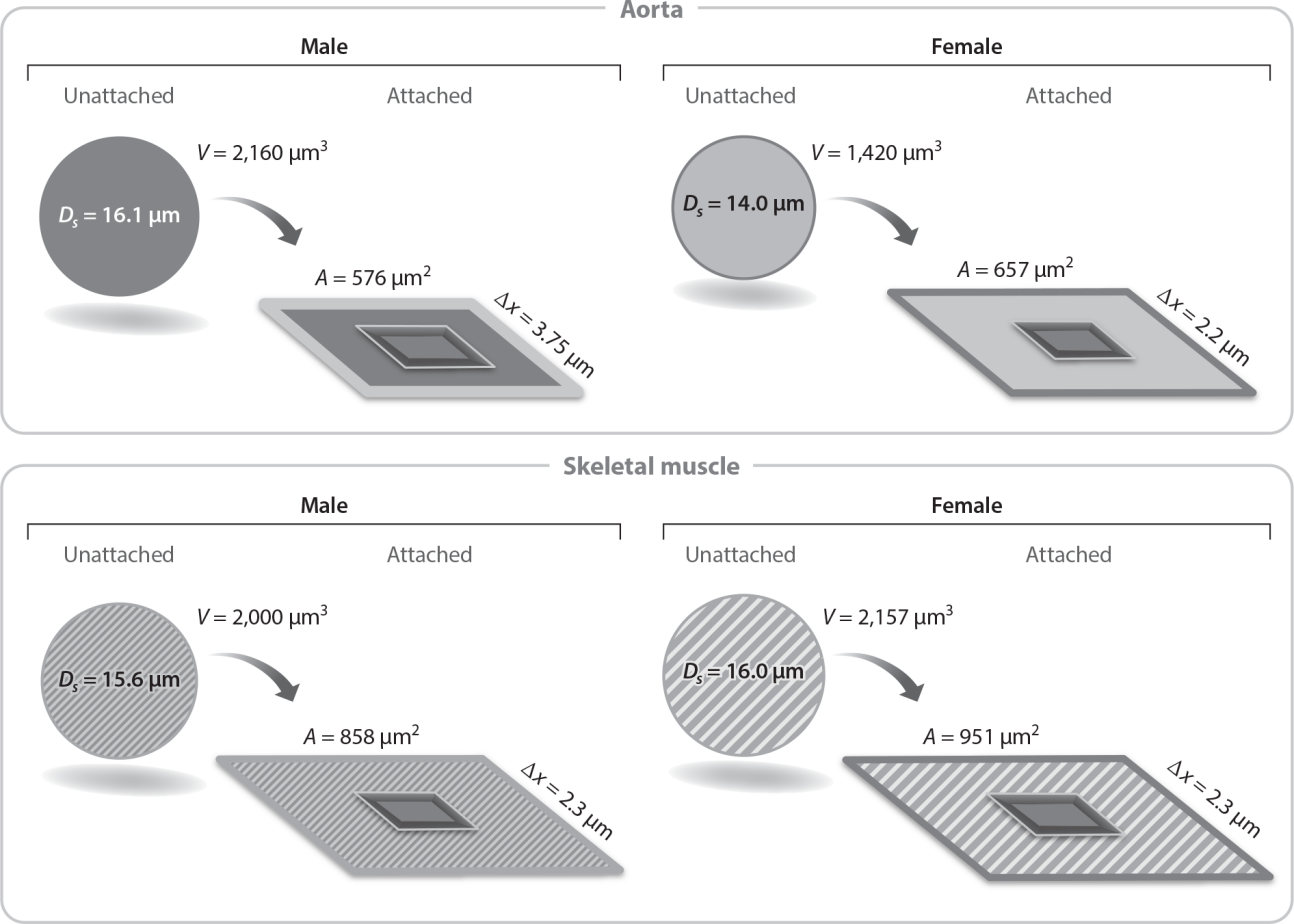
Illustration of the implications of sex and tissue differences in endothelial cell volume and spreading on endothelial cell thickness, Δ*x*, a determinant of barrier permeability. Abbreviations: *V*, volume; *A*, area; *D*_s_, diameter of segment. Figure adapted with permission from Reference 55; copyright 2018 The Physiological Society.

Cellular behavior and function can diverge for male and female cells as well. Often cell morphology is assessed as an insightful feature of cell behavior with practical usefulness and diagnostic relevance (54). Unattached cells can be the same size (55). However, cell spreading can be sex specific upon attaching to a substrate. As shown for endothelial cells, female cells can be larger than male cells under certain substrate conditions and the same size in others (18, 55) (Figure 3). Practically, this can translate to differences in coverage (e.g., re-endothelization of a stent or vascular graft) (55), common experimental end points (e.g., affecting reproducibility, introducing sources of variability) (54), and diagnostic methods (e.g., image analysis and trained machine learning models) (56). Other routine metrics used to evaluate the performance of an engineered tissue are migration and proliferation. Rapid proliferation and migration can indicate faster ex vivo tissue development and patient recovery. In vitro cell proliferation can be sexually dimorphic for human umbilical vein endothelial cells (HUVECs) (F > M), porcine aortic valvular interstitial cells (M > F), and rat oligodendrocyte precursor cells (F > M) (57–59). Additionally, these sex differences can be tissue specific; rat aortic endothelial cells showed no difference in proliferation with sex, while rat microvascular endothelial cells did (M > F) (55). Migration can be similarly sexually dimorphic and tissue specific. Often, cells from the sex with greater proliferative ability migrated faster when challenged by a scratch assay (57, 59). However, this may not always be the case. Female rat aortic endothelial cells migrated faster than those of males, while rat microvascular endothelial cells showed no sex difference in migration rate (55). Cell physical metrics can be sex specific (55, 57–60) and should be appropriately considered.

Complimentary to differences in gene expression, cell proteomes and secretomes can be sexually dimorphic (61–64). Notable proteins often used to evaluate construct efficacy can be sex specific in their expression and activity. For instance, nitric oxide (NO) signaling by embedded endothelial cells is often assessed when evaluating vascular constructs. For HUVECs, female cells produce more transcripts of *NOS3* and its transcribed protein, endothelial nitric oxide synthase (eNOS), and show greater eNOS activation than males (62). Correspondingly, it was shown using in vitro angiogenesis experiments that sprouting was eNOS-dependent for female endothelial cells but not for male cells (62). Similarly, secretion of proangiogenic growth factors (e.g., VEGF, PDGF, bFGF, ET-1) by porcine valvular interstitial cells can be sexually dimorphic and differ with activation state (63). For example, female quiescent valvular interstitial cells produced more VEGF than male cells; however, the sex difference was lost upon activation (63). Activation of these cells was mediated by substrate properties (e.g., stiffness, composition), suggesting regulation and control of these sex differences by microenvironment conditions (63, 65). Many sex differences additional experimentation is needed to understand these behavioral sex differences following stimulation by matrices and signals. Control of the secretome using transgenic cells can be an opportunity for engineering design. Using a hydrogel laden with interleukin (IL)-4 overexpressing mesenchymal stem cells for treating a critical-size bone defect injury in a murine model showed that healing was enhanced for both male and female mice. However, notable sex differences were observed for functional end points, such as the number of macrophages (M > F) and osteoclasts (F > M) at the site of healing (66).

As has been demonstrated by existing literature, SABV impacts cell genetic expression, differentiation, morphology, and function and can manifest at the process engineering level relevant to diagnostics, development, and translation to the clinic. However, cell sex remains a neglected consideration in an alarming number of cell studies. Given the pervasive impact of biological sex on cellular parameters, conferring greater weight to biological sex in experimental design is warranted.

### 2.2. Matrices and Scaffolds

Cells are only one component of engineered tissue. The acellular matrix in which the cells reside, i.e., the scaffold, must provide the necessary architecture and stimuli to elicit desired cell phenotypes and clinical outcomes. Regardless of material type (natural or synthetic), the objective of the scaffold matrix is to engineer a microenvironment with the appropriate chemical, physical, and mechanical properties of the target tissue to promote regeneration.

In recent years, it has been recognized that tissue composition can be sexually dimorphic (67–70). A large body of work has focused on the extracellular matrix (ECM) composition of aortic valves due, in part, to the clinically recognized sex difference in the presentation of fibrocalcific aortic valve disease; more often, males present with calcification and females with fibrosis (70–72). Histological and computed tomographic analyses revealed that female valves had greater collagen content and less calcification density than male valves (72). As for valvular calcification, Gourgas et al. (69) characterized mineral deposits of surgically excised calcified valves and found sex differences in mineral composition and morphology, suggesting that minerals formed more slowly in female valves and by a different mineralization pathway. Using a degradable hydrogel ECM mimic, Schroeder et al. (73) showed that extracellular osteopontin mitigated calcification by female valvular interstitial cells and that elevated amounts of extracellular osteopontin could reduce calcification by male valvular interstitial cells. Elevated amounts of osteopontin in female valve tissues were suggested as a potential sex-specific mitigator of valvular calcification during disease progression (73). In vitro work has shown that matrix remodeling can also be sexually dimorphic, owing to differences in ECM and matrix metalloproteinase production (74, 75). Evidence suggests that such sex-specific cell–substrate interactions may be more generic across cell types. Transcriptomic analyses of endothelial cells and proteomic analyses of iPSC-derived smooth muscle cells have revealed sex-based differences in cell–substrate adhesion pathways, warranting expanded study of other cell types (47, 76). Descriptions and adoption of sex-specific scaffold compositions are nascent, requiring further research to determine the extent of other tissue-specific sex differences in matrix composition in terms of their main structural components (e.g., collagens), modifiers (e.g., glycosaminoglycans), and minerals (e.g., calcium phosphate and its dopants). Moreover, the biophysical and biochemical mechanisms contributing to sex-specific cell–substrate interactions require further investigation.

The effects of physical cues from the ECM and scaffolds differentiate by sex. Physical cues include a material’s geometric features (e.g., size, shape, porosity, dimensionality). For example, male and female osteoblasts respond similarly to microtopographies; however, upon sex hormone stimulation, they can display substrate-dependent responses (77, 78). Similar implant-mediated responses to hormonal stimuli have been shown in the context of vascular implants. In vivo host response to a tricomponent polyglycolic acid–polycaprolactone–poly-l-lactide vascular graft was sex specific and was impacted by tamoxifen, an estrogen receptor modulator (79). Explanted grafts from females displayed greater cell abundance, collagen amount, and maturity, whereas grafts from males elicited greater cell proliferation (79). No difference in endothelialization was measured; however, females showed greater giant cell formation (~2 times). Additionally, grafts from males were more degraded at the end of the study (79). The cellular response to nanoparticles has displayed sex differences as well (80). Bharadwaj et al. (81) showed in vivo that blood–brain barrier permeability to circulating nanoparticles was heightened in female mice compared with male mice following traumatic brain injury. Serpooshan et al. (51) showed in vitro that the uptake of nanoparticles by human amniotic stem cells was greater in female cells than in male cells. At the same time, the reverse was found for human salivary gland fibroblasts. The extent to which scaffolds’ physical features (e.g., porosity and dimensionality) yield sexually dimorphic behavior remains to be determined. The few studies that have reported sex differences to physical cues suggest their significant role in determining cell fate, warranting their consideration in scaffold design.

Cell phenotype is, in part, driven not only by chemical and physical cues but also by the mechanical cues of the cellular microenvironment. Considerable progress has been made in showing the contribution of matrix mechanical properties on cell phenotype and stem cell differentiation (82–84). Only recently has substrate (and matrix) viscoelasticity been shown to support sexually dimorphic phenotypes. Aguado et al. (65) demonstrated that valvular interstitial cell activation was sex specific in response to soft and stiff substrates. In addition, male and female cells can display sex differences in response to mechanical deformation (e.g., shear stress, stretching, and pressure). The endothelial cell response to shear stress is well established; this cell type aligns and elongates in the stress direction and adopts a phenotype protective against atherosclerosis (85). Yet, Lorenz et al. (86) showed that male and female endothelial cells respond differently at the transcriptomic level. Upon stimulation by shear stress when cultured on glass substrates, female endothelial cells differentially expressed hundreds more genes than male endothelial cells (86). To better capture the in vivo microenvironment, James & Allen (18) stimulated male and female endothelial cells with combinations of physiologically relevant levels of shear stress and substrate stiffness. Previous work had reported synergistic and antagonistic responses to such combinations, but not in the context of biological sex (85). James & Allen mapped endothelial cell response using a factorial design of experiments and found combinations of stimuli that gave sexually dimorphic phenotypes and others that did not (18). In particular, nuclear localization of Yes-associated protein 1 (YAP1) differentiated by sex with respect to mechanical stimulation. Such findings underscore that mechanics can modulate phenotypic sex differences. Cellular response to hydrostatic pressure can be sexually dimorphic as well. Ma et al. (87) investigated the effect of cyclic hydrostatic pressure and simulated microgravity on meniscal fibrochondrocytes using a type I collagen scaffold. Few sex differences were observed for ECM protein production, expression of target genes (e.g., *SOX9*), and scaffold mechanical properties, findings which were partially attributed to significant intra-sex donor variability. Notably, Pearson’s correlation analyses of these measurables potentiated a sex-dependent difference in cytoskeletal activity in response to mechanical stimulation, further identified by transcriptomic profiling (87).

Collectively, across several tissues and cell types, there is growing evidence that mechanically activated cell behavior can be sexually dimorphic, the mechanotransduction mechanisms of which deserve further investigation. To realize patient-specific tissue engineered constructs will require expanding our limited knowledge about the sex-specific response to matrix properties.

### 2.3. Signals

In addition to cells and scaffolds, signals complete the tissue engineering triad. Signals can include various natural hormones, biochemical markers, signal transduction components, and pharmacological agents. One goal of manipulating chemical signaling in tissue engineering and regenerative medicine is to mediate and control cellular processes such as differentiation, inflammation, and vascularization. Biological sex influences these processes through signaling molecules (e.g., sex hormones) and biological machinery (88, 89).

The production and metabolism of endogenous molecules can be sex specific. A significant class of such molecules, particularly related to biological sex, includes the three major types of sex hormones: androgens, estrogens, and progestogens. All three types of sex hormones are present in male and female serum; however, they differ in concentration as a function of biological sex and age. Additionally, for females, sex hormone levels depend on pregnancy status and stage of menopause. These compounds are often associated with sexual development and reproductive function, but they also pervade many other aspects of health and disease, such as inflammation and metabolism (90, 91). For instance, coadministration of testosterone or dihydrotestosterone with tumor necrosis factor alpha (TNF-α) amplified inflammatory effects in male and female HUVECs, while estradiol did not (92). On its own, estradiol can increase HUVEC ECM attachment, proliferation, migration, and tubulogenesis (93). Estrogen signaling can dictate stem cell behavior (89).The physiological balance of sex hormones is essential for sex-specific tissue development and sexually dimorphic physiological characteristics. Hormone balance is controlled by many factors, including biotransformation enzymes such as aromatase (CYP19A1), which metabolizes testosterone to estrogen. Thus, sex hormone levels are a paramount chemical signal to address when designing therapies for males and females.

Precision medicine concerns patient traits (e.g., sex hormone levels) and the patient’s environment, which can impact sex hormone signaling. Patients are exposed to a wide range of chemicals used in everyday products, many of which are endocrine disruptors (e.g., phthalates and bisphenols) (94, 95). For instance, these chemicals are present in beauty products and affect males and females differently by being activators or inhibitors of estrogenic and androgenic signaling (96). Thus, in developing tissue engineered systems and regenerative medicine therapies, it is necessary to consider not only endogenous sex hormones but also a patient’s environmental exposures to endocrine disrupting chemicals, among others (e.g., obesogens and neurotoxicants), when developing courses of personalized treatment. Considering environmental chemical exposures in therapeutic design at the preclinical stage may help alleviate clinical translation challenges at later stages of therapy development.

The expression and action of endogenous, nonhormonal small molecules can be sexually dimorphic. NO is an endogenous small molecule well known for its functioning to preserve cardiovascular health, influence the immune response, and modulate liver regeneration; these attributes have contributed to its use as a therapeutic (97). Thus, recognizing NO’s sexually dimorphic expression and action is of interest when developing personalized treatments. Sex differences in NO synthase expression have been noted at the cellular level, with greater production in female cells (62). In multiple rodent models, female aortic tissue samples were more responsive to NO synthase inhibition (specifically, by N^G^-nitro-l-arginine methyl ester) than male tissue samples (98). Additionally, the bioactivity of NO is mechanosensitive (see Section 2.2), illustrating an interplay between biological sex, chemical signaling, and mechanical signaling that requires further study (18). NO exemplifies how an endogenous, bioactive small molecule can have sexually dimorphic activity, and the possibility for other such molecules to exhibit similar sexual dimorphisms should be considered.

An unresolved challenge for tissue engineering is vascularization (99). Many strategies exist and often leverage VEGF’s chemotactic effect of directing endothelial cell migration and stimulating them toward an angiogenic phenotype (100). Yet, often unrecognized is that the endothelial cell response to VEGF is sexually dimorphic. For instance, upon treatment with VEGF following starvation, female HUVECs demonstrated greater migration and intracellular ATP and citric acid cycle metabolite levels than males (100). From this observation, Lorenz et al. (100) proposed that female HUVECs have an increased stress tolerance and energetic advantage in low-nutrient conditions compared with males. Similarly, female HUVECs displayed greater survival, less oxidative stress, and less impaired angiogenesis under hyperoxic conditions than males (101). This study noted that male HUVECs expressed more VEGF receptor 2 mRNA than females under standard culture conditions; however, this difference was not observed at the protein level. Other work involving long-term serum starvation suggests greater adaptation potential of female cells than male cells, owing to their sex-specific secretome (102). Given the criticality of nutrient supply for most tissue engineering applications, these data support considering the role of biological sex in vascularization.

Additionally, cytokines and other inflammatory markers (e.g., TNF-α, IL-10) can differ in their expression between males and females (103). For instance, male peripheral blood mononuclear cells have been reported to produce more TNF-α following lipopolysaccharide (LPS) exposure (104) and more IL-10 from Toll-like receptor 9 (TLR9) activation (105). Greater TNF-α expression from LPS treatment was also seen in whole male blood (106). Another study reported significantly higher circulating levels of IL-1β, IL-6, and TNF-α in male patients, all of which can act as proatherogenic cytokines (107). Numerous aspects of the immune response demonstrate sexual dimorphism, seemingly due to either chromosomal (108) or hormonal (109) differences. As immunoengineering expands, recognizing sex differences and designing for them will be necessary for clinical translation.

Given the prolific use of small molecules, growth factors, and cytokines for therapeutic signaling, as well as the delivery of exogenous pharmaceuticals in tissue engineering and regenerative medicine, the role of biological sex and sex hormones in metabolic processing is a requisite topic to explore. In addition to other considerations, pharmacological solutions must be tailored to sex-specific pharmacodynamic and pharmacokinetic processes. Clinically, sex differences in the absorption, distribution, metabolism, and elimination of drugs should inform drug selection and dosage used in tissue engineering efforts (110). The overall bioavailability of administered drugs depends on these differences. As such, consideration of patient sex can be essential to ensure effective dosage via the appropriate administration route while avoiding toxicity and adverse effects. In their review, Zucker & Prendergast (16) found that sex-based differences in drug pharmacokinetics corresponded to similar differences in adverse drug reactions for 88% of the drugs they evaluated. Sex-specific pathophysiology is a significant consideration for cell and scaffold drug delivery platforms for women’s health applications (111).

Differences in absorption based on sex have been reported in the literature for decades, including intramuscular and oral administration routes (112). In addition, several variables that demonstrate sex-based differences can affect drug distribution within the body, including body mass index, total body water, body fat percentage, plasma volume, and organ perfusion (113, 114). These factors can impact the distribution of drugs within the target tissue and circulation, resulting in different concentration-dependent responses. Notably, the differences in total body water (generally higher in males than females) and body fat percentage (generally higher in females than males) can affect the volume of distribution of drugs, depending on their hydrophilicity. That is, females have a greater volume of distribution for lipophilic drugs and a lesser volume for hydrophilic drugs (115). Another consideration is plasma binding proteins affecting free drug concentration in the bloodstream (114), such as albumin, which tends to bind acidic drugs, and alpha-1 acid glycoprotein (AAG), which tends to bind basic drugs (116). Estrogens have been reported to increase the amount of serum-binding proteins (117), implying a potential sex difference by virtue of sex-specific estrogen levels.

The metabolism of drugs adds another layer of complexity to the sex differences observed for drug pharmacokinetics. Drug metabolism predominantly occurs in the liver, as well as in the kidneys, gastrointestinal tract, lungs, and skin (110, 114). One of the major families of metabolizing enzymes in the liver is cytochrome P450. These enzymes are responsible for the biotransformation of biotic and xenobiotic molecules. Yang et al. (118) conducted a sex-based genetic expression analysis of hepatic drug metabolizing enzymes and transporters (DMET), finding a greater than 1.5-fold change in human female sex-biased expression of several cytochrome P450 genes (*CYP3A4*, *CYP3A7*, *CYP7A1*) and other DMET genes. Previous work has also noted the greater expression of CYP3A4 in female liver tissue (119). Fadiran & Zhang (115) noted that increased estrogen and progesterone concentrations could impact liver enzymatic activity, increasing the accumulation or decreasing the elimination of certain drugs.

Excretion of pharmacoactive substances and their metabolites occurs via several routes to clear them from the body. The kidneys are the primary site of elimination, and sex differences have been noted for renal clearance. Glomerular filtration, tubular secretion, and tubular reabsorption have sex-based differences, generally resulting in greater renal clearance in males than in females (110). Naturally, renal pathology can impact excretory function and, therefore, pharmacokinetics. Sex hormone concentration has also been associated with the excretion of certain chemicals. For instance, Galloway et al. (120) reported increased bisphenol A urine excretion with increasing total testosterone concentration in men. Biliary excretion, the major extrarenal route of elimination, is also noted to demonstrate sex differences (e.g., more efficient excretion of tartrazine and aldosterone in female rats compared with male rats) (121). Altogether, it is essential to actively consider the potential differences in absorption, distribution, metabolism, and excretion between male and female patients. Such considerations may take the form of sex-specific immunomodulatory regimens to mitigate rejection or changes in the drug-loading concentrations in constructs to achieve the desired bioavailability and pharmacokinetic profile of therapeutic agent(s) to maximize efficacy.

For tissue engineering and regenerative medicine, it is crucial to consider the pharmacokinetic factors noted above for their pharmacodynamic outcomes regarding drug exposure and toxicity. This is the case for drug-loaded constructs and drug regimens used in combination with tissue engineered products. For example, cardiovascular differences at the cell and systemic levels have been noted. It would be prudent when administering anticoagulants, using drug-eluting stents, or engineering cardiovascular tissues to assess SABV. The differences in drug metabolism and excretion, as well as in adverse events for diuretics, indicate the consideration of sex for liver and kidney cells, tissues, and products. Being attentive to the sex differences of pharmacoactive molecules may also help mitigate the risk of adverse drug reactions, many of which depend on biological sex.

## 3. PRACTICAL ASPECTS OF CELL SEX STUDIES

Several practical considerations exist when incorporating biological sex into regenerative medicine and tissue engineering basic research (23, 24, 122). One of the most immediate considerations for cell-based studies is sourcing sex-specific cells. Depending on the cell type, this can be nontrivial. Cell sourcing is complicated by vendor availability of adequate numbers of distinct donor populations of each biological sex. On the other hand, primary cells isolated from a study population may require more recruited donors. Logistically, a greater number of cell populations can result in greater monetary expense for purchasing multiple populations of cells, cell culture media, and cell culture consumables, as well as a greater time expense for culturing many more populations. Cell culture media formulation also needs to be considered. For example, phenol red can exhibit estrogen-like activity (123, 124); therefore, using phenol red–free media is necessary to mitigate confounding effects. Additionally, sex-specific media formulations are needed to isolate the effects of sex hormones or replicate their physiologically relevant concentrations. Some more common sex hormones added to cell culture media are dihydrotestosterone (DHT) and 17β-estradiol (E2), among other androgens and estrogens. Formulations for sex-based studies may also require using a defined medium, charcoal-stripped serum, or human-derived serum (23). Before experimentation, it is also critical to validate the sex of the cells to ensure their reported sex is correct and, for male cells, that they have not lost their Y chromosome. Unfortunately, many commercial vendors do not provide sufficient information regarding the sex of their cells (125), especially for cell lines (126). Polymerase chain reaction can be used as a straightforward method to validate the sex of cell populations (127).Assessing sex differences requires intentionality and organization in experimental design to reduce confounding effects, including subject selection, sex-specific disease incidence, the interaction of sex differences with age, reproduction cycles and stages, sample size and randomization, pharmacology, culture conditions, and statistical analyses (128).Including SABV in cell studies is critical to pursuing meaningful avenues of future research toward developing more personalized and effective medical therapies. Correspondingly, the omission of SABV on the foundational level of cell experiments can lead to incomplete or inaccurate conclusions for follow-up translational and clinical studies.

## 4. TRANSLATION OF PRECLINICAL RESEARCH TO CLINICAL STUDIES

Sex is one component of many patient-specific traits considered by precision medicine. The NIH mandates the inclusion of women, as well as minorities and subjects from young to old (20). Consideration of these variables is critical because other demographic factors such as age (129) and ancestry can interact with biological sex. For example, in the context of sex hormones, males experience a decrease in serum testosterone levels with advancing age, and postmenopausal females experience a decrease in serum estrogen and androgen levels. These reductions are associated with several physiological changes and increased morbidities (130). Additionally, estrogen and androgen differences have been reported for females and males of different ancestry (131, 132). Pregnancy status in women also affects sex hormone levels (133) and other parts of physiological homeostasis, including immune regulation (134).

Precision medicine considers not only patient physiology but also a patient’s environment and lifestyle. For instance, environmental chemical exposures to toxicants can disrupt basal endocrine signaling (among other signaling). In another example, engineered tissues implanted in mobile animals (i.e., the recovering tissue receives significant mechanical stimulation) may not translate to patients exhibiting more sedentary lifestyles, possibly resulting in poorer patient outcomes. Maternal and neonatal health can also be influenced by environmental exposures, ancestry, and other sociodemographic variables (94). Thus, a patient-specific treatment should not be designed in a vacuum and needs to consider the interactions of biological sex with environmental conditions and lifestyle choices.

Rich-Edwards et al. (128) provide a comprehensive tabulation of best practices for clinical study methodology regarding motivation, sample selection and size, data collection and analysis, and reporting and interpretation. Additionally, they note that not all statistically significant differences seen for in vitro studies translate to meaningful differences in vivo, with nontrivial and clinically relevant magnitudes. Similarly, Wallach et al. (135) noted in their meta-analysis that few sex–treatment interactions met the threshold for clinical statistical significance. Furthermore, it is essential to recognize the sex bias still prevalent in numerous clinical studies and to design in vivo experiments that genuinely represent the target patient population. Female underrepresentation in clinical studies for several diseases, such as cardiovascular and chronic kidney diseases, has been documented (136). Compounding on this, Mirin (137) notes a disparity in disease research funding, biased in favor of male-dominated diseases. Additionally, pregnant individuals have historically been excluded from clinical trials (138). Reasons cited for this intentional exclusion include the vulnerability of the pregnant individual, altered pharmacokinetics, and the risks of adverse effects to the fetus. Practically, this exclusion shifts the development of treatments from controlled research settings to the less certain clinic (139). When translational studies have included pregnancy, identifying and enrolling pregnant individuals can be difficult because of complicated and inconsistent categorical search criteria (e.g., investigational versus previously FDA-approved interventions, pregnancy stage targeted, and benefit intended for the pregnant woman or the fetus) (140). Engaging prenatal health providers and community-based organizations has been proposed as a strategy for addressing these challenges (141). At all levels, researchers designing regenerative medicine therapies and engineered tissue systems must be mindful of robust experimental design methodology and the broader context of the patients they serve.

## 5. LOOKING FORWARD AND CHALLENGES AHEAD

Recently, strategies have been put forth to guide researchers on properly including SABV in basic and clinical research to assuage concerns regarding the misreporting of sex-based differences due to improper analysis methods and misinterpretation of results (142, 143). Given the deepening interest in this topic, another challenge is the need to train researchers to test for and report sex differences. Toward this end, the NIH provides several resources, including training modules, administrative funding supplements, and a center program focused on sex differences (22). However, wholly incorporating SABV to meet the objectives of tissue engineering and regenerative medicine requires expanding upon the shift in scientific culture that is beginning to take hold (144, 145). All entities involved in the conduct of research have a responsibility to further sex equity, including accurate vendor reporting of cell sex, parity in clinical trial representation and treatment by clinicians, equal funding of disease research by agencies, and inclusion of sex in experimental design by researchers.

Despite the advantages of including SABV in biomedical engineering and regenerative medicine research, some, including ourselves, believe that this is one step in the right direction but does not and will not adequately address health disparities. To advance from regenerative medicine to precision regenerative medicine, we believe that researchers must consider the complex milieu of biological variables—sex, gender, age, genetic ancestry, and health status—as well as incorporate environmental, lifestyle, and social and communal factors. It is by accounting for the intersection of these many variables that the most individualized therapeutic approaches can be provided.
